# Brain sex differences: the androgynous brain is advantageous for mental health and well-being

**DOI:** 10.1038/s41386-021-01141-z

**Published:** 2021-08-16

**Authors:** Qiang Luo, Barbara J. Sahakian

**Affiliations:** 1 Institute of Science and Technology for Brain-Inspired Intelligence, Ministry of Education-Key Laboratory of Computational Neuroscience and Brain-Inspired Intelligence, Fudan University, Shanghai, China; 2 National Clinical Research Center for Aging and Medicine at Huashan Hospital, Fudan University, Shanghai, China; 3 Department of Psychiatry, University of Cambridge, Cambridge, UK; 4 Behavioural and Clinical Neuroscience Institute, University of Cambridge, Cambridge, UK

**Keywords:** Cognitive neuroscience, Medical research

Studies of personality and sex differences have reported that male’s and female’s basic personality traits appear to differ on average, in several respects [1]. From a psychological perspective, these sex differences are thought to result from perceived gender roles, gender socialisation and socio-structural power differentials, although the evolutionary theory has also been used to explain these differences [1]. However, psychological studies have already suggested that people can be androgynous, having mixed personality traits that are stereotypically considered to be male or female [2]. Furthermore, most of us are in fact probably somewhere on a spectrum between what we stereotypically consider a male or a female [2].

Importantly, such ‘psychological androgyny’ has long been associated with traits such as better cognitive flexibility (the mental ability to shift between different tasks or thoughts), social competence, mental health [3] and emotional reactivity [4]. For example, extreme masculine norms are detrimental, as evidenced by a meta-analysis showing that conformity to these norms incurs social costs and psychiatric symptoms, including depression, loneliness and substance abuse [5]. In addition, considering DSM-5 diagnoses, autism spectrum disorder, attention-deficit/hyperactivity disorder and conduct disorder are more commonly diagnosed in males, whereas eating disorders, major depressive disorder and generalised anxiety disorder are more commonly diagnosed in females [6]. But how does this relate to the brain? Is there such a thing as ‘brain androgyny’? While male and female brains are similar, the connectivity between brain areas has been shown to differ [3]. We used these connectivity markers to characterise the brains of 9620 participants (4495 male and 5125 female). Indeed, our findings suggest that brain androgyny does exist [3]. Using the sex differences of the resting-state functional connectivity we built a multivariate classifier to estimate the likelihood of a given functional brain network to represent a male brain and achieved 77.75% classification accuracy when the model was applied to an independent dataset. Instead of binarizing the output of this classifier, we created a brain continuum, with male and female at the extreme ends, using the continuous output of this classifier. Indeed, we found that participants who mapped at the centre of this continuum, representing androgyny, had fewer mental health symptoms, such as depression and anxiety, compared with those at the two extreme ends. These findings support our novel hypothesis that there exists a neuroimaging concept of brain androgyny, which may be associated with better mental health in a similar way to psychological androgyny. The complex interaction of biological, psychological and environmental factors and how they modulate brain function requires further understanding. Future research on these topics is needed to progress understanding as well as to elucidate the influences on brain androgyny across the life span. Given that we have found that an androgynous brain offers better mental health, it follows that, for optimal performance in school and work, and for better well-being throughout life, we need to avoid extreme stereotypes and offer children and adolescents opportunities not restricted by their biological sex as they grow up.
