# Correction to: Brain sex differences: the androgynous brain is advantageous for mental health and well-being

**DOI:** 10.1038/s41386-021-01202-3

**Published:** 2021-10-27

**Authors:** Qiang Luo, Barbara J. Sahakian

**Affiliations:** 1 Institute of Science and Technology for Brain-Inspired Intelligence, Ministry of Education—Key Laboratory of Computational Neuroscience and Brain-Inspired Intelligence, Fudan University, Shanghai, China; 2 National Clinical Research Center for Aging and Medicine at Huashan Hospital, Fudan University, Shanghai, China; 3 Department of Psychiatry, University of Cambridge, Cambridge, UK; 4 Behavioural and Clinical Neuroscience Institute, University of Cambridge, Cambridge, UK

**Keywords:** Cognitive neuroscience, Medical research

Correction to: *Neuropsychopharmacology* 10.1038/s41386-021-01141-z, published online 16 August 2021

The article “Brain sex differences: the androgynous brain is advantageous for mental health and well-being”, written by Qiang Luo and Barbara J. Sahakian, was originally published electronically on the publisher’s internet portal on 16 August 2021 without open access. With the author(s)’ decision to opt for Open Choice the copyright of the article changed on 25 August 2021 to © The Authors 2021 and the article is forthwith distributed under a Creative Commons Attribution 4.0 International License, which permits use, sharing, adaptation, distribution and reproduction in any medium or format, as long as you give appropriate credit to the original author(s) and the source, provide a link to the Creative Commons licence, and indicate if changes were made.

The images or other third-party material in this article are included in the article’s Creative Commons licence, unless indicated otherwise in a credit line to the material. If material is not included in the article’s Creative Commons licence and your intended use is not permitted by statutory regulation or exceeds the permitted use, you will need to obtain permission directly from the copyright holder.

To view a copy of this licence, visit http://creativecommons.org/licenses/by/4.0/.

The original article has been corrected.

